# Case Report: Transcatheter treatment of aortic coarctation in a 58-year-old patient with LACHT syndrome and left lung agenesis

**DOI:** 10.3389/fcvm.2023.1239624

**Published:** 2023-11-30

**Authors:** Qingxian Tu, Nanqu Huang, Fujia Guo, Minhong Luo, Min Xu, Jiaji Liu, Zhengqiang Yuan, Qianfeng Jiang

**Affiliations:** Department of Cardiovascular Medicine, Third Affiliated Hospital of Zunyi Medical University (The First People’s Hospital of Zunyi), Zunyi, China

**Keywords:** LACHT syndrome, aortic coarctation, lung agenesis, aorta covered stent, hypertension

## Abstract

LACHT (Lung Agenesis, Congenital Heart, and Thumb anomalies) syndrome is an extremely rare congenital anomaly and presents significant challenges in adults due to its poor survival rates. Herein, we report a case of late diagnosis and successful transcatheter treatment of aortic coarctation in a 58-year-old male patient with LACHT syndrome, medically resistant arterial hypertension, and left lung agenesis. Baseline CT angiography showed isthmic aortic coarctation and left lung agenesis, with compensatory right pulmonary artery and vein thickenings. The patient underwent balloon dilation and subsequent implantation of a covered NuMED 45 mm 8-ZIG CP stent with satisfactory outcomes. The pressure gradient decreased from 43 to 23 mmHg. The arterial pressures normalized during the follow-up with fewer medications. Genetic testing identified a heterozygous mutation (c.6583C > T) in the FBN2, supporting the diagnosis of variant Marfan syndrome.

## Introduction

LACHT (Lung Agenesis, Congenital Heart, and Thumb anomalies) syndrome, also known as the Mardini Nyhana association, is an exceedingly rare and medically serious congenital disorder (1). To date, only 13 documented cases of LACHT syndrome exist worldwide, with one instance leading to prenatal diagnosis and pregnancy termination at 36 weeks (2). Among these cases, only two have reported cardiac malformations, including aortic coarctation (2). Notably, no documented cases of lung agenesis associated with aortic coarctation have survived into adulthood. Furthermore, information on the genetic basis of LACHT syndrome remains limited, with no specific pathogenic gene identified to date. In this report, we present a case of late diagnosis and successful transcatheter treatment of aortic coarctation in a 58-year-old male with LACHT syndrome. Familial genetic testing revealed a heterozygous mutation (c.6583C > T) in FBN2, supporting the diagnosis of a variant of Marfan syndrome.

## Case presentation

**Figure 1:**
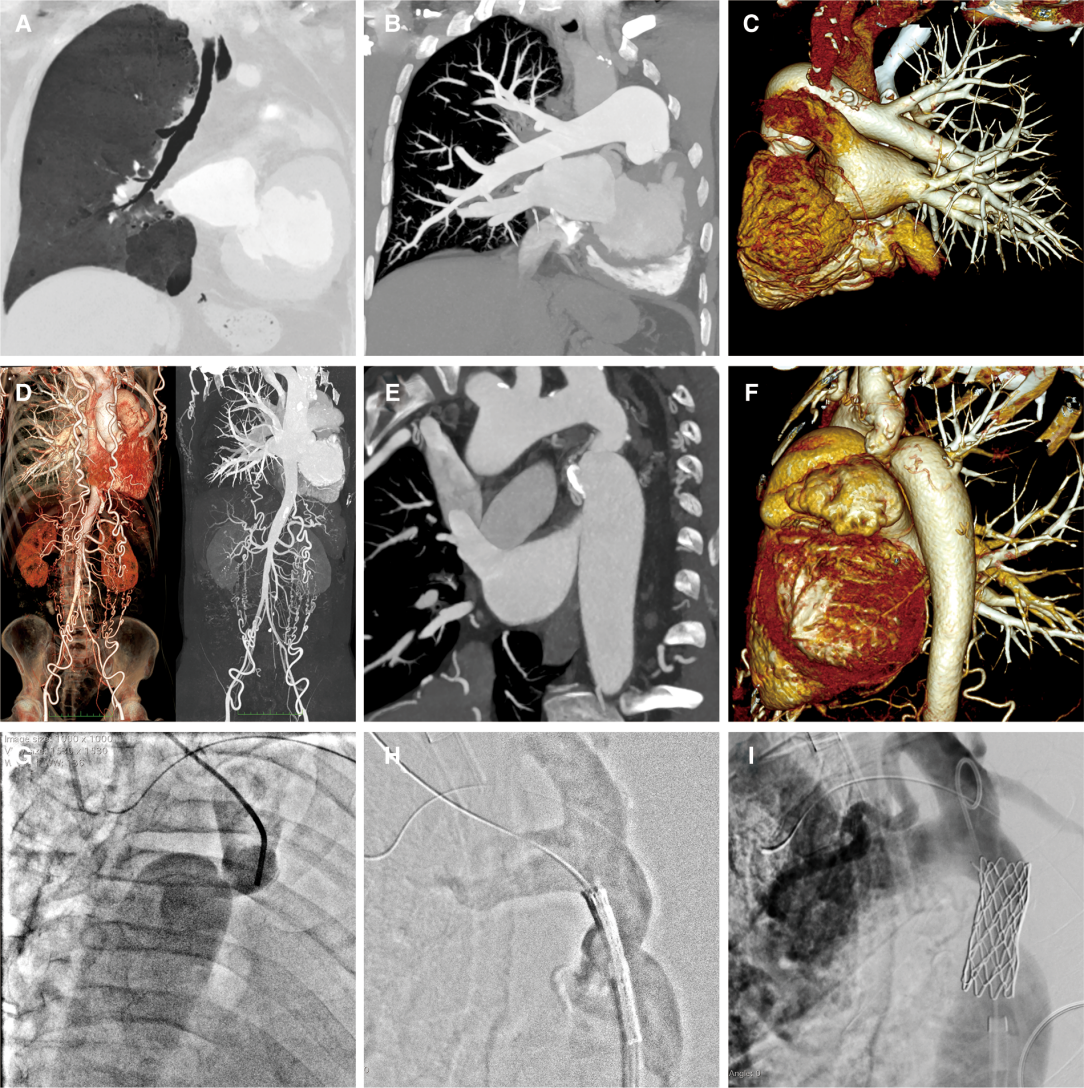
**A-C**: Pulmonary artery CTA reveals the absence of the left lung structure, with no display of the left pulmonary artery and its branches. The right lung structure and pulmonary artery show no filling defect, and the branches of each lobe segment artery are well displayed with compensatory dilatation. **D-F**: Aortic CTA shows localized narrowing at the beginning of the descending aorta and branches are formed at the distal ends of the subclavian artery, intercostal arteries, intrathoracic arteries, and both iliac arteries. **G-I**: Aortic angiography assesses the degree of narrowing, locates the position of the balloon-expandable covered stent, and evaluates the post-release effects.

A 58-year-old male patient was transferred to our institution for rapidly worsening medically-resistant arterial hypertension and shortness of breath. The patient also complained of dizziness and weakness in both lower limbs, with occasional symptoms of blurred and darkening vision, lower limb edema, and syncope. The patient had a history of left pulmonary agenesis that was diagnosed at the age of 8 years old without any further investigation. The patient has been taking antihypertensive drugs irregularly in the past until it was adjusted recently to include amlodipine 5 mg once daily, Irbesartan 150 mg once daily, Metoprolol 12.5 mg twice daily, hydrochlorothiazide 12.5 mg once daily, and Terazosin 2 mg once nightly. One month after adjusting the medication, the blood pressure remained uncontrolled. The patient had a body mass index of 20.5 kg/m^2^, was not a smoker, and reported moderate alcohol consumption. Family history showed that his mother had mild arterial hypertension. Physical examination showed an arm-leg systolic pressure gradient of 40 mmHg and a grade 2/6 vascular murmur in the left scapular area. Physical abnormalities included funnel chest, mild collapse of the left chest wall, and the absence of breathing sounds in the left lung. Upon admission, serum creatinine, homocysteine, B-type natriuretic peptide, and low-density lipoprotein cholesterol were mildly elevated, while thyroid function and blood sugar were normal. Echocardiography showed a left atrium diameter of 43 mm, left ventricle diameter of 56 mm, interventricular septum thickness of 12 mm, left ventricular posterior wall thickness of 12 mm, and left ventricular ejection fraction of 61%. Computed tomographic thoracic imaging was performed and revealed left pulmonary agenesis (Figures 1A–C) along with a severe isthmic coarctation of the aorta just distal to the dilated left subclavian artery. There was abundant collateral circulation bypassing the coarctation in the suprascapular, internal thoracic, and superficial abdominal arteries (Figures 1D–F). 24 h ambulatory blood pressure monitoring showed non-dipper hypertension. Pulmonary function tests showed mixed ventilation dysfunction with predominant moderate to severe obstruction. Renin, angiotensin, and aldosterone levels were within the normal range at three-time points (0:00, 8:00, 16:00) in both standing and lying positions. The rheumatoid factor test showed no specific positive indicators. Carotid artery ultrasound, renal artery ultrasound, and adrenal CT scan showed no significant abnormalities. A coronary angiogram showed no significant stenosis in the left anterior descending artery or circumflex artery, and an aneurysmal-like dilation at the opening of the right coronary artery.

**Figure 2:**
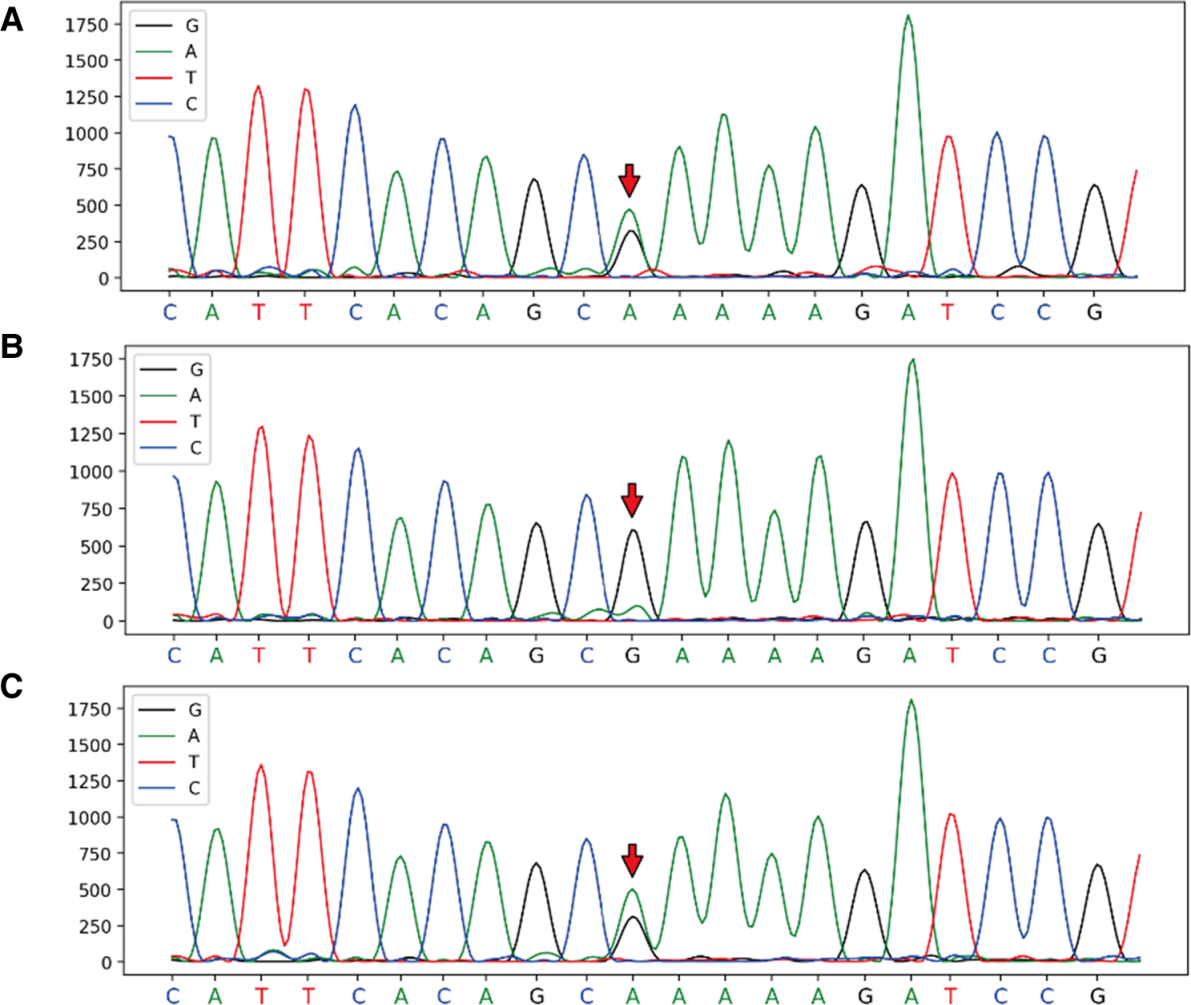
Full exon gene sequencing first generation sequencing family verification chart verification site: FBN2, c.6583C > T, chromosome position: chr5:127624873. (**A**) Proband: heterozygous mutation at chr5:127624873; (**B**) Mother: wild-type at chr5:127624873; (**C**) Son: heterozygous mutation at chr5:127624873.

With the consent of the patient and family, and approval from the hospital ethics committee, a whole-exome sequencing family genetic screening was performed, and revealed a heterozygous mutation in FBN2, c.6583C > T supporting the diagnosis of variant Marfan syndrome, with the mother being wild-type and her son showing the heterozygous mutation (Figure 2).

A multidisciplinary decision was made to treat this patient with transcatheter stent implantation under general anesthesia. The left femoral vein was accessed to place a temporary cardiac pacemaker. The right radial arterial access was then obtained to measure the pressures and perform invasive angiography. A 14F Cook sheath was introduced through the right femoral artery, followed by aortic dilation with a Fortrex 6 × 40 mm balloon. Subsequently, a 45 mm covered 8-Zig CP-stent (NuMED, USA) was mounted on a 13 × 40 mm balloon and implanted in the initial segment of the descending aorta (Figures 1G–I). Angiography confirmed the safe adhesion and secure placement of the stent. There was a significant reduction in the pressure gradient from 43 to 23 mmHg.

Post-procedural monitoring did not reveal any abnormalities in renal function. A repeat aortic CT angiography was performed one-month post-procedure and showed stable results. Eighteen months post-procedure, the patient's blood pressure remained stable within the normal range under two antihypertensive drugs, and limb symmetry was restored. A 6 min walking test of 530 meters was conducted. There was no change in echocardiography findings compared to hospitalization, and the left ventricular ejection fraction was within the normal range.

## Discussion

LACHT syndrome, or Mardini-Nyhan association, is an extremely rare disorder and is characterized by complex cardiac vascular malformations and skeletal abnormalities (2, 3). Pulmonary agenesis is a rare congenital developmental defect and is classified into three types according to the complete or partial absence of the lung parenchyma, bronchus, and pulmonary artery (1, 4). Nearly half of the cases of pulmonary agenesis are accompanied by other congenital abnormalities affecting various systems (2). The clinical manifestations of pulmonary agenesis can vary from asymptomatic cases to respiratory conditions such as dyspnea and respiratory distress. Our patient presented with type 1 pulmonary agenesis (absence of lung parenchyma, bronchus, and pulmonary artery) and was diagnosed with chronic obstructive pulmonary disease.

Coarctation of the aorta is a congenital condition that accounts for 5% to 10% of all congenital vascular malformations (5). Marfan syndrome (MFS) is an autosomal dominant connective tissue disorder that affects the ocular, skeletal, and cardiovascular systems. Aortic tear and rupture are the leading causes of death in individuals with MFS. In our patient, family gene screening revealed a heterozygous mutation at the FBN2 gene, specifically c.6583C > T. This mutation is consistent with the inheritance pattern of a dominant disease (6) and has been associated with a variant Marfan syndrome (7).

Although the diagnosis of aortic coarctation is not challenging, it can be overlooked, particularly in elderly patients who may be diagnosed solely with essential hypertension without undergoing comprehensive and meticulous examinations. The patient's delayed diagnosis, survival, and absence of significant target organ damage can be attributed to the development of the aorta with marked collateral circulation (8). The patient suffered from congenital hypoplasia of the left pulmonary vein, resulting in compensatory dilation of the right pulmonary vein and blood draining into the left atrium. We hypothesize that the increase in left atrial volume can be compensated, resulting in a compensatory increase in left ventricular volume. This situation leads to the volume load of the left ventricle entering the aorta, which can also be compensated. Consequently, the aortic vessels experience vascular remodeling due to increased pressure load caused by aortic constriction. Simultaneously, compensatory collateral circulation forms at the distal end of the aortic constriction segment, mitigating vascular remodeling and ischemic tissue damage caused by volume and pressure load. Thus, the initial rise in blood pressure is not substantial, and organ damage remains within a compensatory range. Patients display mild ischemic symptoms and signs exclusively, and the progression of hypertension-related organ damage is relatively slow.

The treatment options for aortic coarctation include surgical and transcatheter repair (9, 10). The patient has one lung agenesis, and the risk of surgical anesthesia was high. We considered treating his coarctation percutaneously with a covered stent to be less invasive and aggressive. However, the patient presented a significant aortic dilation near the proximal end of the constriction with a substantial difference in diameter compared to the distal narrowed portion. Furthermore, the constriction was located at a bend in the vessel, making interventional treatment challenging. We considered the stent length and balloon diameter according to baseline anatomy using the standard approach (10). Following expansion, the stent adhered well to the vessel wall at the level of the narrowing segment. Despite some procedural and technical advantages of other new-generation endovascular stents (11), we have selected the CP stent because of the availability in the armamentarium and operators' experience with CP stents. Even though the residual pressure gradient was not optimal, significant control over blood pressure was achieved with medications. We refrained from further expansion or additional stent placement to avoid the risk of vessel tearing due to excessive expansion (11). Subsequent follow-up showed that the patient's blood pressure was normalized with medication, supporting the correctness of our comprehensive decision.

## Conclusion

We present the case of a 58-year-old male patient with LACHT syndrome with a late diagnosis and treatment of aortic coarctation. The patient underwent successful aortic balloon angioplasty and stent implantation, resulting in better control of normal blood pressure and symptom relief. Genetic testing revealed a variation in the FBN2 gene 253 (c.6583C > T), supporting the diagnosis of variant Marfan syndrome.

## Data Availability

The original contributions presented in the study are included in the article/Supplementary Material, further inquiries can be directed to the corresponding author/s.
